# Evaluation of the Cytotoxicity of Cationic Polymers on Glioblastoma Cancer Stem Cells

**DOI:** 10.3390/jfb14010017

**Published:** 2022-12-28

**Authors:** Conor McCartin, Juliette Blumberger, Candice Dussouillez, Patricia Fernandez de Larrinoa, Monique Dontenwill, Christel Herold-Mende, Philippe Lavalle, Béatrice Heurtault, Stéphane Bellemin-Laponnaz, Sylvie Fournel, Antoine Kichler

**Affiliations:** 1 3Bio Team, Faculté de Pharmacie, CAMB UMR7199 CNRS−University of Strasbourg, 74 route du Rhin, F-67401 Illkirch, France; 2 Institut de Physique et Chimie des Matériaux de Strasbourg (IPCMS) UMR7504, Université de Strasbourg & CNRS 23 rue du Loess, F-67083 Strasbourg, France; 3 Laboratoire de Bioimagerie et Pathologies UMR CNRS 7021 (LBP), Faculté de Pharmacie, 74 route du Rhin, F-67401 Illkirch, France; 4 Division of Neurosurgical Research, Department of Neurosurgery, University of Heidelberg, 69120 Heidelberg, Germany; 5 Institut National de la Santé et de la Recherche Médicale, Inserm UMR_S 1121 Biomaterials and Bioengineering, F-67085 Strasbourg, France

**Keywords:** glioblastoma, cancer stem cells, cationic polymers, polyethylenimine, cytotoxicity

## Abstract

Cationic polymers such as polyethylenimine (PEI) have found a pervasive place in laboratories across the world as gene delivery agents. However, their applications are not limited to this role, having found a place as delivery agents for drugs, in complexes known as polymer-drug conjugates (PDCs). Yet a potentially underexplored domain of research is in their inherent potential as anti-cancer therapeutic agents, which has been indicated by several studies. Even more interesting is the recent observation that certain polycations may present a significantly greater toxicity towards the clinically important cancer stem cell (CSC) niche than towards more differentiated bulk tumour cells. These cells, which possess the stem-like characteristics of self-renewal and differentiation, are highly implicated in cancer drug resistance, tumour recurrence and poor clinical prognosis. The search for compounds which may target and eliminate these cells is thus of great research interest. As such, the observation in our previous study on a PEI-based PDC which showed a considerably higher toxicity of PEI towards glioblastoma CSCs (GSCs) than on more differentiated glioma (U87) cells led us to investigate other cationic polymers for a similar effect. The evaluation of the toxicity of a range of different types of polycations, and an investigation into the potential source of GSC’s sensitivity to such compounds is thus described.

## 1. Introduction

Cationic polymers have found many uses, including as flocculants in wastewater treatment [1], and perhaps most prominently as delivery agents for genetic material to mammalian cells through their ability to form polyelectrolyte complexes (polyplexes) with anionic nucleic acids [2,3]. However, a lesser explored aspect of polycations is their potential as anti-cancer compounds. The investigation of their effects in vivo has seemingly been carried out with restraint following studies showing their interaction with blood serum proteins and the induction of haemolysis [4,5,6]. However, such negative effects have been shown to be reduced by the shielding or reduction in the positive charges of the polymer through chemical modification (e.g., by coupling with PEG (polyethyleneglycol)) or complexation with polyanions [4,7,8,9,10,11,12]. Polycations have thus found a use in pharmaceutical research as delivery systems in the form of polymer-drug conjugates (PDCs) [13,14]. Such conjugation of drugs to polymeric carriers allows for the modification of their properties such as their pharmacokinetics, immunogenicity, and solubility, as well as opening up the realm of targeted drug delivery through linkers which are cleavable under specific conditions (heat, light, etc.) [13]. However, their potential medical applications may not be limited to the role of carrier, with concerns about their in vivo toxicity being potentially overemphasised. A 2005 study by Dufès et al. has shown an inherent capacity of several naked polycations (polypropylenimine dendrimer, linear PEI, and fractured PAMAM dendrimer) to reduce growth and induce tumour regression in a nude mouse xenograft model, without any apparent toxicity to the mice [15]. Chitosan has also shown anti-tumour effects [16,17]. Some studies link this effect to the stimulation of an immune response towards cancer cells rather than purely a direct action of the polymer [17,18]. The observation that cancer cells may be more negatively charged than non-cancerous cells also provides a potential explanation as to why they may be more sensitive to their effects [17,19,20,21]. The precise mechanisms of polycation toxicity have yet to be fully elucidated, but in vitro studies have implied a quick necrosis/membrane permeabilisation linked to a hydrolysis of phospholipids. Reports regarding the induction of apoptosis are conflicting [22,23,24].

Very recently, two studies have indicated a potentially high level of sensitivity of cancer stem cells (CSCs) to the toxicity of cationic polymers [25,26]. CSCs are a type of cancer cell possessing the stem-like characteristics of self-renewal and differentiation which are purported to be at the heart of tumour initiation and recurrence [27,28]. Their role in tumour recurrence is strongly linked to their observed resistance to chemotherapy, which is driven by multiple characteristics such as reprogrammed metabolism, protective autophagy, and upregulated DNA repair mechanisms [29]. Among the cancers where these populations have been identified as being the most prevalent is the highly aggressive brain cancer glioblastoma [30]. Our previous study into the effect of a PEI-based platinum PDC as well as of “naked” PEI on glioblastoma CSC (GSC) cell lines revealed what seemed to be a significantly higher level of toxicity of both compounds on the GSCs compared to non-stem glioma cells (the U87-MG cell line) [31]. This seemed to occur through a necrosis accompanied by an induction of protective autophagy, which is an autophagic response to some chemotherapeutic insults, the inhibition of which may increase the sensitivity of the cells to the treatment [32]. In light of this result, and the two other studies which report a significant toxicity of PEI-coated nanoparticles [25] and a cationic phosphorous dendrimer [26] towards GSCs, we wished to elaborate this potentially important phenomenon. Thus, in this study, we evaluated the cytotoxicity of a range of cationic polymers varying in their size, type and topology towards the NCH421K (GSC) and U87-MG (non-stem glioma) cell lines.

## 2. Materials and Methods

### 2.1. Storage of Compounds

Linear-polyethylenimine 22 kDa (L-PEI) (from Stéphane Bellemin-Laponnaz Laboratory, IPCMS Strasbourg), 25 kDa branched-PEI (B-PEI) (#408727, Sigma-Aldrich, St. Louis, MO, USA), Poly-L-Lysine hydrochloride (#P9404, Sigma-Aldrich), Poly-(Lys,Trp) (#P-9285, Sigma-Aldrich), Poly-L-ornithine hydrobromide (#P3655, Sigma-Aldrich), DGL-NH_2_G4 (Colcom). Poly-Arginines (pArg) of 10, 30, 70 and 200 (#000-R010-102, #000-R030-104, #000-R070-101, #000-R200-102) were obtained from Alamanda Polymers. Poly-L-Arginine 120 (#P7762, Sigma-Aldrich), 0.7 kDa B-PEI (#40871-9, Sigma-Aldrich), 1.8 kDa B-PEI (#40528, Alfa AESAR), 2 kDa B-PEI (#408700, Sigma-Aldrich), 750 kDa B-PEI (#18,197-8, Sigma-Aldrich), 0.73 kDa L-PEI (obtained from Luc Lebeau laboratory, Strasbourg) and 4 kDa L-PEI (#24885-2, Polysciences). Linear polyethylenimine HCl, Mw 4000 (L-PEI HCl 4000) is a fully hydrolysed (deacylated), highly water-soluble hydrochloride salt form of the Linear polyethylenimine of MW 2500. All polymers were resuspended in sterile water and kept at −20 °C.

### 2.2. Cell Culture and Treatment Conditions

Cell culture and treatment conditions of the U87, NCH421K and NCH644 cell lines were previously described [31]. The CHO-K1 and pgsA-745 cell lines were obtained from Dr Eric Vivès (Laboratoire des Défenses Antivirales et Antitumorales, CNRS-UMR5124, Institut de Génétique Moléculaire, Montpellier) [33]. Both cell lines were cultured in RPMI 1640 medium (#R2405-500ML, Sigma-Aldrich, Roswell Park Memorial Institute) medium containing 10% (*v/v*) FBS (#10270106, Gibco, USA), Penicillin–Streptomycin (P/S: 10 U/0.1 mg).

### 2.3. Cell Viability Assay

Cell culture viability measurements were carried out as described in [31] (using the CellTiterGlo 3D Cell Viability Assay (#G9681, Promega). Briefly, following treatment with the desired concentration and timepoint, the reagent was added at a 1:1 ratio to the wells. The wells were vigorously mixed, incubated for 30 min at RT, transferred to an opaque plate and luminescence was then measured using the Safas Monaco. Viability was calculated according to the non-treated control which was considered to be of a 100% viability.

### 2.4. Synthesis of 22 kDa L-PEI and Rhodamine–PEI Conjugate

The 22 kDa L-PEI used for conjugation to rhodamine was synthesised as described previously by [34] with modifications. Briefly, 8 g of poly(2-ethyl-2-oxazoline) (Sigma-Aldrich, #25805-17-8) were hydrolysed with 100 mL of concentrated HCl (37%) and refluxed for 48 h, yielding a white precipitate. The solid was then filtered by vacuum through a sinter glass and washed several times with water. The resulting L-PEI hydrochloride salt was air-dried overnight, dissolved in 50 mL of water and freeze-dried. The L-PEI salt was made alkaline by adding aqueous NaOH (3 M) and the resulting white precipitate was filtered and washed with water until the pH became neutral. The white solid was then dissolved in water and further lyophilized until a white solid was obtained (1.8 g, 71.9%). The polymer was stored dry under argon. Proton nuclear magnetic resonance (^1^H NMR) (400 MHz, CDCl_3_) δ 1.72 (s, 1H, NH), 2.71 (s, 4H, CH_2_CH_2_) ppm (Figure S1).

The synthesis of the PEI–rhodamine conjugate was adapted from [35]. Briefly, under argon, 50 mg of 22 kDa L-PEI (1 eq.) and 3.7 mg of RBITC (3 eq.) (Rhodamine B isothiocyanate, Sigma-Aldrich #283924) were solubilised in 7 mL anhydrous dimethylformamide (DMF) and stirred for 24 h at room temperature. The polymer was precipitated using ice cold diethyl ether and was washed multiple times with diethyl ether until the supernatant became clear in order to eliminate the excess of RBITC. A pink solid was obtained (49.4 mg, 96.45%). The polymer–fluorophore conjugate was stored dry or as a 1 mM stock in absolute ethanol. ^1^H NMR (300 MHz, CDCl3) δ 1.62 (s, 3H, 3xNH), 2.72 (s, 4H, CH_2_CH_2_) ppm (see Figure S2).

### 2.5. Cationic Polymer Affinity Assay

#### 2.5.1. U87 and NCH421K Cells

As a way of measuring the affinity of the cells for cationic non-stem glioma and glioblastoma cancer stem, cells were incubated with a fluorescently tagged cationic polymer at 4 °C (to inhibit endocytosis) to measure its level of cell surface adhesion via flow cytometry. The U87-MG and NCH421K cells were detached and dissociated using Accutase^®^ treatment for 5 min at room temperature before centrifugation (5 min at 350× *g*, 4 °C) and then resuspended in 1 mL cold DPBS containing 3.3 µg/mL Poly-L-lysine (PLL)–FITC conjugate (mol wt 15,000–30,000, P3543, Sigma-Aldrich) or rhodamine–PEI conjugate for a final cell concentration of 2 × 10^5^ cells/mL. The suspension was incubated protected from light on ice for 60 or 20 min (for FITC-PLL and Rhodamine-PEI, respectively) before centrifugation (5 min at 350 × *g*, 4 °C), resuspension in 200 µL DPBS and analysis directly on a flow cytometer (FACSCalibur, Becton Dickinson) in the FL1 channel for the PLL-FITC labelled cells or in the PE channel of a FACSCanto (Becton Dickinson) for the Rhodamine–PEI labelled cells. Fluorescence histograms were quantified and analysed using FlowJo software.

#### 2.5.2. CHO-K1 and pgsA-745 Cells

CHO-K1 and pgsA-745 cells were seeded at 40,000 and 45,000 cells per well, respectively, one day prior to treatment. Medium was aspirated and replaced by 250 µL RPMI 0% FBS, to which 12.5 µL/well of 0.4 mM Rhodamine–PEI (10 µL at 1 mM in ethanol stock added to 15 µL 150 mM NaCl) was added for a final concentration of 18.8 µM. This was left to incubate for 2 h in a 37 °C incubator. The treatment was then removed and replaced by 500 µL RPMI 10% FBS, and left for another 22 h in a 37 °C incubator. Supernatant was then removed, with the wells washed twice with DPBS before recovery of the cells via trypsinisation and analysis of the cells on the flow cytometer (PE channel of a FACSCanto (Becton Dickinson)).

### 2.6. Heparan Sulfate Expression

In order to measure the level of heparan sulphate expression on the NCH421K and U87 cells, 1 × 10^6^ cells/mL in 100 µL were incubated for 30 min on ice in DPBS (#D8537-500ML, Sigma-Aldrich) (2% FBS, #10270106, Gibco) with anti-heparan sulphate antibody (#370255-S, Amsbio) diluted 1/100. The cells were then centrifuged (5 min at 350× *g*, 4 °C) and re-suspended in DPBS to wash excess antibody. The cells were then again centrifuged and resuspended in 100 µL Goat anti-Mouse IgM Secondary Antibody, FITC (ThermoFisher #31992) diluted 1/100 in DPBS (2% FBS) and incubated for 30 min on ice. The cells were then centrifuged once more and resuspended in DPBS (2% FBS) and passed directly on a FACS-Calibur (Becton Dickinson) in the FL1 channel. Fluorescence histograms were quantified and analysed using FlowJo software.

For the CHO K1 and pgs-A745 cells, the protocol was the following: cells were seeded at 200,000 cells per wells one day prior to treatment in a 6-well plate. The next day, the medium was aspirated and the cells washed with DPBS. The cells were then detached using accutase mixed with DPBS (1:1) for 5 min on ice before centrifugation at 350× *g* for 5 min (4 °C). The cells were then stained similarly to the U87 and NCH421K cells.

## 3. Results and Discussion

### 3.1. GSC Specific Toxicity Is Not a General Characteristic of Polycations

**Figure 1:**
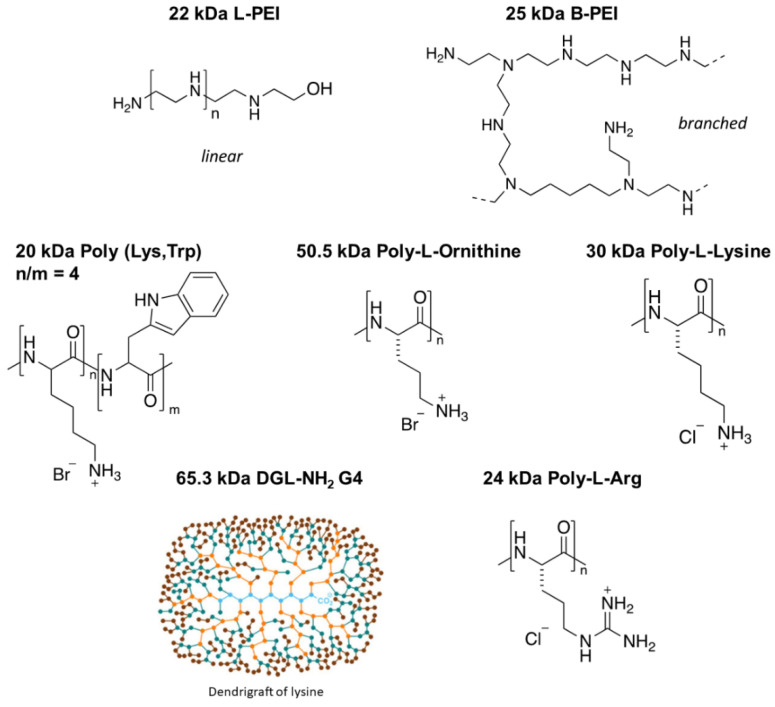
Structures of the tested polymers. A total of 22 kDa linear polyethylenimine (L-PEI), 25 kDa branched PEI (B-PEI), 20 kDa Poly(Lys,Trp) 4:1 hydrobromide, 50.5 kDa Poly-L-ornithine (PL-Orn) hydrobromide, 30 kDa Poly-L-lysine hydrochloride (PLL), dendrigrafts of lysine of generation 4 (DGL-NH_2_G4) with a MW of 65.3 kDa [38], and 24 kDa Poly-arginine hydrochloride (PL-Arg).

As a GSC model, the NCH421K cell line was used. This cell line, isolated from a patient suffering from glioblastoma, was maintained in a stem-like phenotype through growth in serum-free “stem cell” medium [36,37]. The natural in vitro growth morphology of these cells under such conditions was a non-adherent spheroid morphology (Figure S3). This cell line was to be compared to the more differentiated U87-MG glioma cell line. The toxicity of an array of cationic polymers on the NCH421K GSCs and the U87-MG cells was thus assessed via the ATP-based CellTiter-Glo^®^ 3D Cell Viability Assay in order to assess whether they potentially possessed a higher level of toxicity towards GSCs. The polycations with MW ranging from 22 to 65.3 kDa differing in their type as well as their topology (branched vs. linear) (Figure 1) were assessed in terms of their 50% viability inhibitory concentrations (IC_50_) with respect to the concentration of polymer (Table 1; Figures S4 and S5).

As shown in Table 1, all the polymers were cytotoxic for the GSC cell line NCH421K with IC50 ranging from 133.3 nM for the most toxic one to 620 nM for the least toxic polymer. To note, toxicity of the polymers is not restricted to the NCH421K cell line since we previously reported that L-PEI 22 kDa had an IC50 of 624 nM when using the GSC cell line NCH644 [31]. In addition, here, we show that the polylysine of 30 kDa has an IC50 of 558 nM ± 206 on NCH644 (Figure S6), a value that is similar to that found for NCH421K.

**Figure 2:**
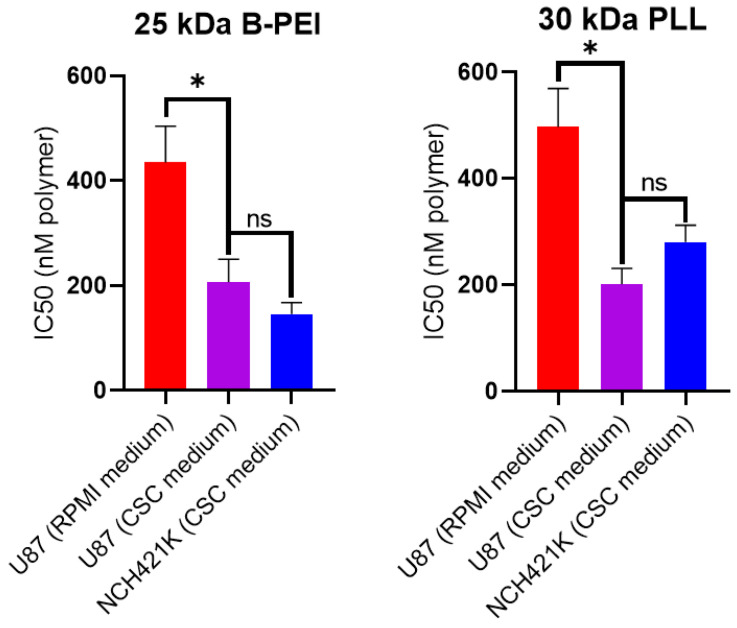
Effect of CSC medium on 25 kDa B-PEI and 30 kDa PLL toxicity on U87-MG cells. Histograms showing 24 h IC50 (calculated from CellTiterGlo 3D viability measurements by non-linear regression using Prism GraphPad 8 software) values of 25 kDa B-PEI and 30 kDa PLL (expressed in nM of polymer) on U87-MG cells seeded in their standard medium (RPMI 10% FBS) and then treated in either their standard medium (RPMI 10% FBS) or in serum-free CSC medium. Values represent the mean of at least *n* = 3 independent experiments ± one SEM. Statistics represent Student’s *t*-tests. ns = *p* > 0.05. * = *p* ≤ 0.05. Distribution normality was confirmed using a Shapiro–Wilk test.

The result showed a significant difference in toxicity between each of the polymers with a roughly 2- to 3.5-fold greater toxicity towards the NCH421K GSCs compared to the U87 cells. The exception was the 22 kDa L-PEI, which showed an eight-fold difference, as recently reported by [31]. Importantly, this latter study identified a potentially significant difference in toxicity linked to the difference in culture medium between the two cell lines (serum-free DMEM/F12 (“CSC medium”) for GSCs and 10% serum RPMI for U87). This manifested as a roughly 800 nM decrease in IC_50_ of 22 kDa L-PEI on U87 cells treated in CSC medium (i.e., an IC50 of 1547 nM in RPMI versus approx. 700 nM in CSC medium). Thus, it is possible that the approximate twofold difference observed between U87 and NCH421K for most polymers (Table 1)—except 22 kDa L-PEI and B-PEI 25 kDa—is due to the medium. To verify this point, we treated U87 cells with 25 kDa B-PEI and the 30 kDa PLL in RPMI and CSC medium. As shown in Figure 2, the IC50 of the two polymers was indeed decreased and the differences between U87 and NCH421K were no longer significant.

We thus reiterate and emphasise our previous warning regarding the comparison of toxicities between cell lines with vastly different culture media [31], which is especially pertinent in the CSC domain due to the media required for the maintenance of their stem-like phenotype [39,40,41,42]. Although, at least in this case, the phenomenon seems to be restricted to cationic polymers (or at least to not be generally applicable to all molecules), as an iridium compound previously assessed for its action as an anti-CSC therapeutic by our team did not display a culture medium-dependent change in toxicity on U87 cells (Figure S7) [39]. The effect of CSC-specific toxicity thus appeared to be a 22 kDa L-PEI-specific effect rather than a general effect applicable to all polycations. The identification of a source for this specificity is thus a major point of interest.

### 3.2. GSC Polycation Affinity Is Not Linked to Heparan Sulfate Expression

**Figure 3:**
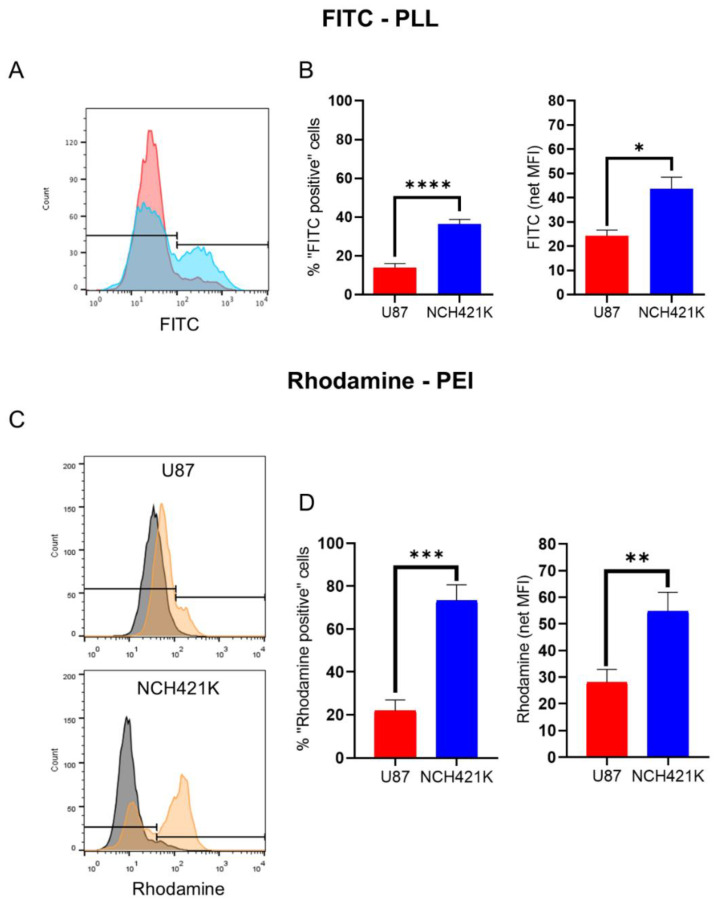
U87-MG vs. NCH421K cationic polymer affinity. (**A**) Representative fluorescence histograms showing U87-MG (red) and NCH421K (blue) FITC fluorescence following a 1 h incubation at 4 °C with FITC-conjugated PLL and the set positive/negative gate (fluorescence of non-treated cells was not detectable). (**B**) Histograms of FITC-PLL fluorescence of U87 and NCH421K cells expressed as positive/negative gating (left) as shown in A and via net geometric mean fluorescence intensity (MFI stained—MFI non-treated) (right). Values represent the mean of at least *n* = 3 independent experiments ± one SEM. Statistics represent Student’s *T*-tests. ns = *p* > 0.05, * = *p* ≤ 0.05, **** = *p* ≤ 0.0001. Distribution normality was confirmed using a Shapiro–Wilk test. (**C**) Representative fluorescence histograms (FlowJo) showing rhodamine fluorescence following a 20 min incubation at 4 °C with rhodamine conjugated PEI with the set positive/negative gating. Black = Non-treated. Orange = Rhodamine–PEI treated. (**D**) Histograms of Rhodamine–PEI fluorescence difference between U87 and NCH421K cells expressed as positive/negative gating (left) shown in C, and via net geometric mean fluorescence intensity (MFI stained—MFI non-treated) of Rhodamine–PEI-incubated U87 and NCH421K cells. Values represent the mean of at least *n* = 3 independent experiments ± one standard error of the mean (SEM). Statistics represent Student’s T-tests. ** = *p* ≤ 0.01. *** = *p* ≤ 0.001. Distribution normality was confirmed using a Shapiro–Wilk test.

In order to investigate why the toxicity of 22 kDa L-PEI was significantly higher on the NCH421K cells than on the differentiated U87 glioma cells (even accounting for the effect brought by differences in culture media), we wished to measure the affinity of the cells for fluorescent polycations. This was carried out by incubation of the cells with fluorophore (FITC)-conjugated PLL (15–30 kDa) and rhodamine-conjugated 22 kDa L-PEI at 4 °C in order to limit endocytosis and instead only observe electrostatic binding to the cell surface. The result for the FITC-PLL conjugate was an unexpected bimodal distribution (Figure 3A), with the NCH421K cells showing a significantly higher proportion of FITC-positive cells and a higher calculated fluorescence intensity than the U87 cells, indicating a higher affinity for the polymer (Figure 3B). This was also the case for the rhodamine–PEI conjugate, for which a bi-modal distribution was less evident on the U87 cells than for the FITC-PLL conjugate (Figure 3C), but for which there was a significant difference between the U87 and NCH421K cells calculated both in terms of fluorescence positive/negative populations and by net fluorescence intensity (Figure 3D).

As affinity for cationic polymers was hypothesised to be linked to a higher negative cell surface charge, and heparan sulphate glycoproteins are known to significantly contribute to the negative membrane charge of mammalian cells [43] as well as being shown to be implicated in the mode of entry of PEI and polylysines into cells [44,45,46], we wished to compare the heparan sulphate expression of the two cell lines via flow cytometry with an antibody raised against heparan sulphate proteoglycan.

**Figure 4:**
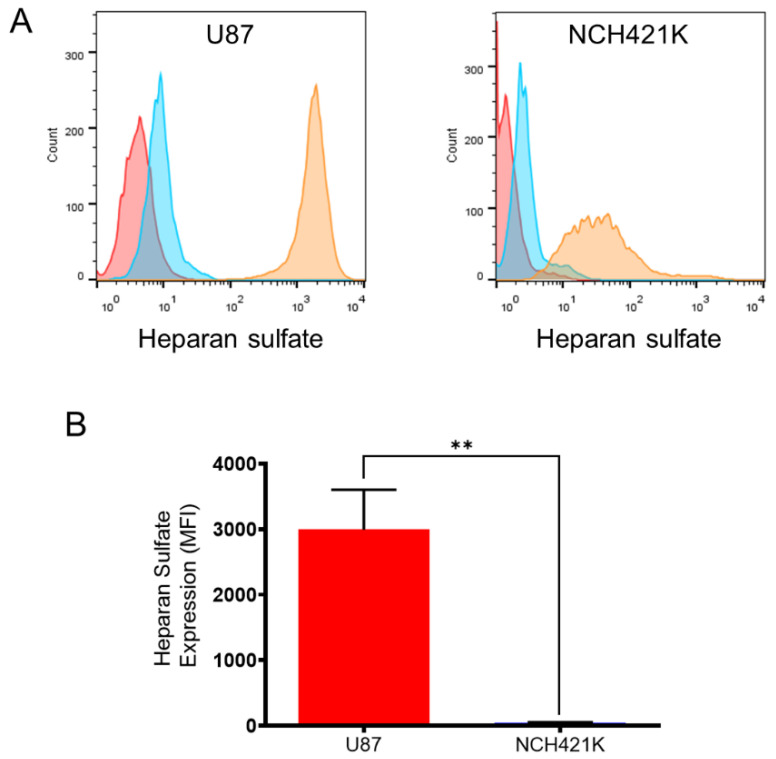
U87-MG vs. NCH421K heparan sulphate expression. (**A**) Representative fluorescence histograms (FlowJo) showing U87-MG (left) and NCH421K (right) heparan sulphate expression. Red = Non-marked cells. Blue = Secondary antibody only. Orange = Primary + secondary antibody. (**B**) Histograms representing net median fluorescence intensity (MFI) (MFI secondary antibody only—MFI primary + secondary) of heparan sulphate expression on U87-MG and NCH421K cells. Values represent the mean of at least *n* = 3 independent experiments ± one SEM. Statistics represent Student’s *t*-tests. ns = *p* > 0.05. ** = *p* ≤ 0.01.

Inversely to the polycation affinity, the result showed that the U87 cells had a considerably higher expression of heparan sulphate compared to the NCH421K cells (Figure 4), which is consistent with studies showing high expression of heparan sulphate on glioma cells [47,48,49]. This observation is of interest with regards to the established link between increased heparan sulphate sulphation levels and stem cell differentiation [50,51], although increased levels of the heparan sulphate proteoglycan syndecan 1 have been associated with CSC characteristics [52].

The higher sensitivity of the NCH421K cells towards 22 kDa L-PEI despite the vastly lower heparan sulphate expression is especially interesting considering the implication of heparan sulphate in PEI adsorption/entry, which we displayed by the lower rhodamine–PEI signal on the glycosaminoglycan deficient CHO-pgsA-745 mutant [53] compared to the wild type (Figure S8). This suggests an as of yet unidentified source of affinity and toxic sensitivity of GSCs towards 22 kDa L-PEI.

### 3.3. GSC Polycation Toxicity Is Size-Dependent

Otherwise, we wished to investigate the effect of varying molecular weight on the toxicity of cationic polymers on the NCH421K cells. The result showed a significant increase in toxicity with the molecular weight for PL-Arg (Table 2; Figure S9), with the toxicity increasing almost proportionally with the number of monomer units.

Varying molecular weights of L-PEI were also tested (Table 3 and Figure S10), with the result showing again an increase in toxicity with increased size of the polymer.

This is in accordance with previous studies which have shown a similar size-dependent toxicity on tumor cells of PEI [24,54,55] and PLL [24,56]. To note, the fold difference in toxicity of 4 kDa L-PEI between the IC_50_ values for the U87 and NCH421K cell lines was not as great as for the 22 kDa L-PEI. However, it indicates that the relevant sensitivity towards 22 kDa L-PEI is partly maintained for its smaller counterpart. The results thus confirm that while it is not the case for all cationic polymers, GSCs seem to have a high level of sensitivity to the toxicity of L-PEI, which is maintained for different lengths of the polymer.

## 4. Conclusions and Outlook

This work is a follow-on study which showed GSCs to present a specific sensitivity to the toxicity of 22 kDa L-PEI, which induced a necrosis-like, autophagy-inducing cell death with characteristics of immunogenic cell death [31]. Considering this and two recent reports in the literature which also indicated that GSCs were sensitive to polycation toxicity [25,26], we wished to investigate whether the observed effect was a phenomenon general to polycations. The results showed a higher level of toxicity towards GSCs than towards non-stem glioma cells for all of the polycations tested. However, it seems that in making such a comparison, polycations appear more toxic on GSCs than on non-stem glioma cells due to the differences in their culture media. This is a significant observation which should be carefully noted in the domain of in vitro anti-CSC therapeutics research. Nevertheless, in spite of this, an important sensitivity towards 22 kDa L-PEI remains apparent, a sensitivity which may be linked to a higher affinity of the GSCs towards a fluorescent L-PEI conjugate, and which was in spite of a very low expression of heparan sulphate, a cell surface motif which has been established as important in the mode of entry of polycations into mammalian cells [44,45,46]. Of note, however, is the fact that although the GSCs did display a higher affinity towards a fluorescent PLL conjugate, the IC50 between CSC and U87 cells were similar (when using GSCs culture medium for both cell lines). Separately, a size dependence of PL-Arg and L-PEI toxicity was shown. These results provide some clarity and direction regarding the relationship of GSCs and polycation toxicity. While L-PEI remains interesting and warrants further investigation as a potential CSC therapeutic, considerable work must be conducted to understand why GSCs are sensitive to this compound and to establish whether the effect is specific to GSCs, or an even more interesting phenomenon common to CSCs of different tissue origin. A further array of polymers should be tested to search for others with a similar effect, hopefully narrowing down the chemical characteristics responsible. Should a causal link be established, polymers may find themselves unexpectedly at the heart of a highly important therapeutic search.

## Figures and Tables

**Table 1:** IC_50_ values of various polycations tested on U87 and NCH421K cell lines.

POLYMERS	IC_50_ ± SEM (nM Polymer)		
[Approx. Number of Monomers/Molecule]	U87	NCH421K	
22 kDa L-PEI [523]	1547 ± 206	****	194 ± 23.5
25 kDa B-PEI [595]	508.8 ± 12.3	***	146.2 ± 12.9
30 kDa PLL [182]	583.4 ± 17.1	**	280.5 ± 18.2
20 kDa P-(Lys,Trp) [164]	514.9 ± 9.7	**	257.1 ± 17.3
50.5 kDa PL-Orn [259]	344.2 ± 26.7	*	133.3 ± 5.1
65.3 kDa DGL-NH_2_G4 [365]	558.8 ± 16.5	*	292.2 ± 35.9
24 kDa PL-Arg [120]	1570 ± 85	Ns	620 ± 55

All IC_50_ values are the mean of at least three independent experiments ± one standard error of the mean (SEM), with each experiment consisting of the mean of at least three independent replicates. IC50 values were calculated from CellTiterGlo 3D viability measurements by non-linear regression using Prism GraphPad 8 software. Statistics (calculated using Prism) represent Student’s *T*-tests. ns = *p* > 0.05. * = *p* ≤ 0.05. ** *p* ≤ 0.01. *** = *p* ≤ 0.001. **** = *p* ≤ 0.0001. Distribution normality was confirmed using a Shapiro–Wilk test. L-PEI = Linear PEI. B-PEI = Branched PEI. PLL = Poly-L-Lysine. P-(Lys,Trp) = Poly-Lysine-Tryptophan. PL-Orn = Poly-L-Ornithine. PL-Arg = Poly-L-Arginine. DGL-NH2G4 = Dendrigrafts of Lysine of generation 4. In brackets are the approximative numbers of monomers per molecule.

**Table 2:** IC_50_ values of PL-Arg of varying size on NCH421K cells.

Poly-Arginine [Monomers/Molecule]	IC_50_ ± SEM (nM Polymer)
1.9 kDa PL-Arg [10]	>8000
5.8 kDa PL-Arg [30]	2380 ± 64
13 kDa PL-Arg [70]	890 ± 98 ****
24 kDa PL-Arg [120]	620 ± 64 *
38.5 kDa PL-Arg [200]	460 ± 75 ^ns^

All IC_50_ values are the mean of at least three independent experiments ± SEM, with each experiment consisting of the mean of at least three independent replicates. IC_50_ values were calculated from CellTiterGlo 3D viability measurements by non-linear regression using Prism GraphPad 8 software. Statistics (calculated using Prism) represent Mann–Whitney tests between the value on which the star is indicated, and the value of the next lowest molecular weight PL-Arg. ns = *p* > 0.05. * = *p* ≤ 0.05. **** = *p* ≤ 0.0001. PL-Arg = Poly-L-Arginine hydrochloride. In brackets are the approximative number of monomers per molecule.

**Table 3:** IC_50_ values of L-PEI of varying size tested on U87 and NCH421K cell lines.

Polyethylenimine	IC_50_ ± SEM (nM PEI)		
[Monomers/Molecule]	U87		NCH421K
0.73 kDa L-PEI [17]	ND	/	>32,000
4 kDa L-PEI [60]	3337.2 ± 178.8	****	1285 ± 188.7
22 kDa L-PEI [523]	1547 ± 205.9 ^****^	****	194 ± 23.5 ****

All IC_50_ values are the mean of at least three independent experiments ± SEM, with each experiment consisting of the mean of at least three independent replicates. IC_50_ values were calculated from CellTiterGlo 3D viability measurements by non-linear regression using Prism GraphPad 8 software. Statistics (calculated using Prism) superscript to values represent Student’s T tests between the value on which the stars are indicated, and the value of the next lowest molecular weight. Statistics in between values represent Student’s *T* tests. **** = *p* ≤ 0.0001. Distribution normality was confirmed using a Shapiro–Wilk test. In brackets are the approximative content number of monomers (EI, Ethylenimine) per molecule.

## Data Availability

Data are contained within the article or Supplementary Materials. Raw data may be made available upon request.

## Supplementary Materials

The following supporting information can be downloaded at: https://www.mdpi.com/article/10.3390/jfb14010017/s1, Figure S1. 1H NMR spectrum of synthesised 22 kDa L-PEI. 400 MHz, CDCl3; Figure S2. Reaction schematic and 1H NMR spectrum of synthesised 22 kDa L-PEI. 300 MHz, CDCl3; Figure S3. NCH421K spheroid cell culture. Inverted light microscope image of NCH421K spheroids grown in 96 well culture dishes for 4 days before imaging at 4× * 10× = 400× magnification using an Axio Vert A1 inverted light microscope (Zeiss) coupled to a ProgRes C5 cool (Jenoptik) camera; Figure S4. IC50s for the U87-MG cell line. CelltiterGlo 3D viability dose-response of 24 h treated U87 cells. Values represent the mean of at least n = 3 independent replicates. Error bars represent +/− one SEM; Figure S5. IC50s for the GSC cell line NCH421K. CelltiterGlo 3D viability dose-response of 24 h treated NCH421K cells. Values represent the mean of at least n = 3 independent replicates. Error bars represent +/− one SEM; Figure S6. IC50s for the GSC cell line NCH644. CelltiterGlo 3D viability dose-response of 24 h treated NCH644 cells. Values represent the mean of at least n = 3 independent replicates. Error bars represent +/− one SEM; Figure S7. Lack of culture medium dependent toxicity for NHC-Ir(III). CelltiterGlo 3D viability dose-response of 24 h treated U87 cells in either their normal (RPMI) or CSC medium. Values represent the mean of at least n = 3 independent replicates. Error bars represent +/− one SEM; Figure S8. Reduced PEI affinity of glycosaminoglycan deficient CHO cells. (A) Representative fluorescence histograms (FlowJo) of 2 h, 37 °C Rhodamine-PEI incubated CHOK1 WT and CHO-pgsA-745 mutant cells. (B) Bar charts of net MFI (median) (MFI stained – MFI unstained) of stained cells. Values represent the mean of n = 2 independent experiments +/− one SEM. (C) Representative fluorescence histograms (FlowJo) of heparan sulfate stained CHOK1 WT and CHO-pgsA-745 mutant cells. (D) Bar charts of net MFI (median) (MFI stained – MFI unstained) of stained cells. Values represent one experiment. Red = Non-stained (A) or secondary antibody only (C) stained CHO cells. Green = Non-stained (A) or secondary antibody only (C) stained pgsA-745 cells. Orange = Rhodamine-PEI stained (A) or primary + secondary antibody stained (C) CHO cells. Blue = Rhodamine-PEI stained (A) or primary + secondary antibody stained (C) pgsA-745 cells; Figure S9. IC50s for the GSC cell line NCH421K. CelltiterGlo 3D viability dose-response of 24 h treated NCH421K cells. Values represent the mean of at least n = 3 independent replicates. Error bars represent +/− one SEM; Figure S10. IC50s for the GSC cell line NCH421K. CelltiterGlo 3D viability dose-response of 24 h treated NCH421K cells. Values represent the mean of at least n = 3 independent replicates. Error bars represent +/− one SEM.

## Abbreviations

B-PEI	Branched PEI
CSC	Cancer stem cell
DMEM	Dulbecco’s Modified Eagle Medium
DPBS	Dulbecco’s phosphate-buffered saline
FBS	Fetal bovine serum
GSC	Glioblastoma stem cell
L-PEI	Linear PEI
MFI	Median/Geometric mean fluorescence intensity
PDC	Polymer–drug conjugate
PEI	Polyethylenimine
PLL	Poly-L-lysine
RLU	Relative luminescence unit
RPMI	Roswell Park Memorial Institute
