# Supplementary material for: Evaluation of the Cytotoxicity of Cationic Polymers on Glioblastoma Cancer Stem Cells
Source: J Funct Biomater. 2022 Dec 28;14(1):17. doi: 10.3390/jfb14010017 (PMC9862959; doi:10.3390/jfb14010017)
Supplement: Supplementary file 1 [file jfb-14-00017-s001.zip › jfb-2080315-supplementary.pdf]

Supplementary Information

Evaluation of the Cytotoxicity of Cationic Polymers on Glioblastoma Cancer Stem Cells

Conor McCartin [1,†], Juliette Blumberger [1,†], Candice Dussouillez [1], Patricia Fernandez de Larrinoa [1,2], Monique Dontenwill [3], Christel Herold-Mende [4], Philippe Lavalle [5], Béatrice Heurtault [1], Stéphane Bellemin-Laponnaz [2], Sylvie Fournel [1,*] and Antoine Kichler [1,*]

[1] 3Bio Team, CAMB UMR7199 CNRS−University of Strasbourg, Faculté de Pharmacie, 74 route du Rhin, F-67401 Illkirch, France
[2] Institut de Physique et Chimie des Matériaux de Strasbourg (IPCMS) UMR7504, Université de Strasbourg & CNRS 23 rue du Loess, F-67083 Strasbourg, France
[3] Laboratoire de Bioimagerie et Pathologies UMR CNRS 7021 (LBP), Faculté de Pharmacie, 74 route du Rhin, F-67401 Illkirch, France
[4] Division of Neurosurgical Research, Department of Neurosurgery, University of Heidelberg, 69120 Heidelberg, Germany
[5] Institut National de la Santé et de la Recherche Médicale, Inserm UMR_S 1121 Biomaterials and Bioengineering, F-67085 Strasbourg, France
* Correspondence: s.fournel@unistra.fr (S.F.); kichler@unistra.fr (A.K.)
† These authors contributed equally to this work.

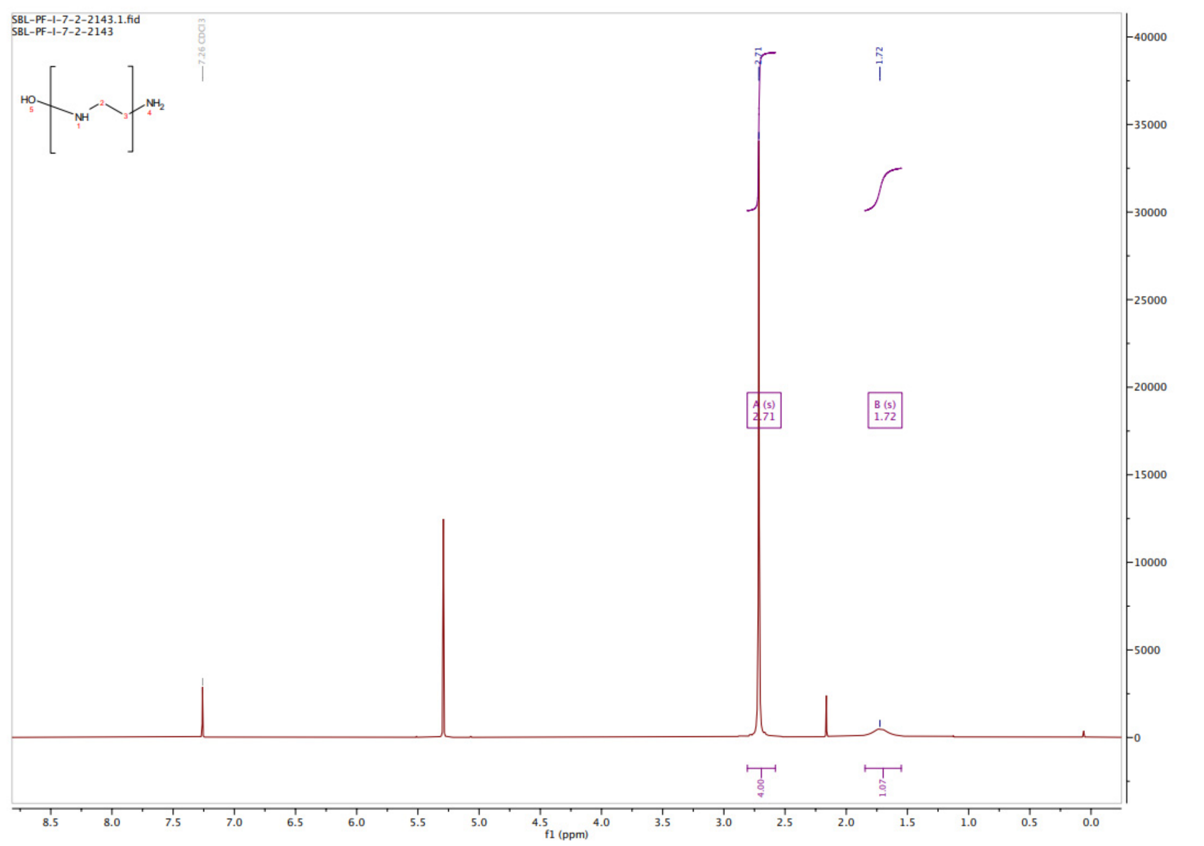

Figure S1. 1H NMR spectrum of synthesised 22 kDa L-PEI. 400 MHz, CDCl3.

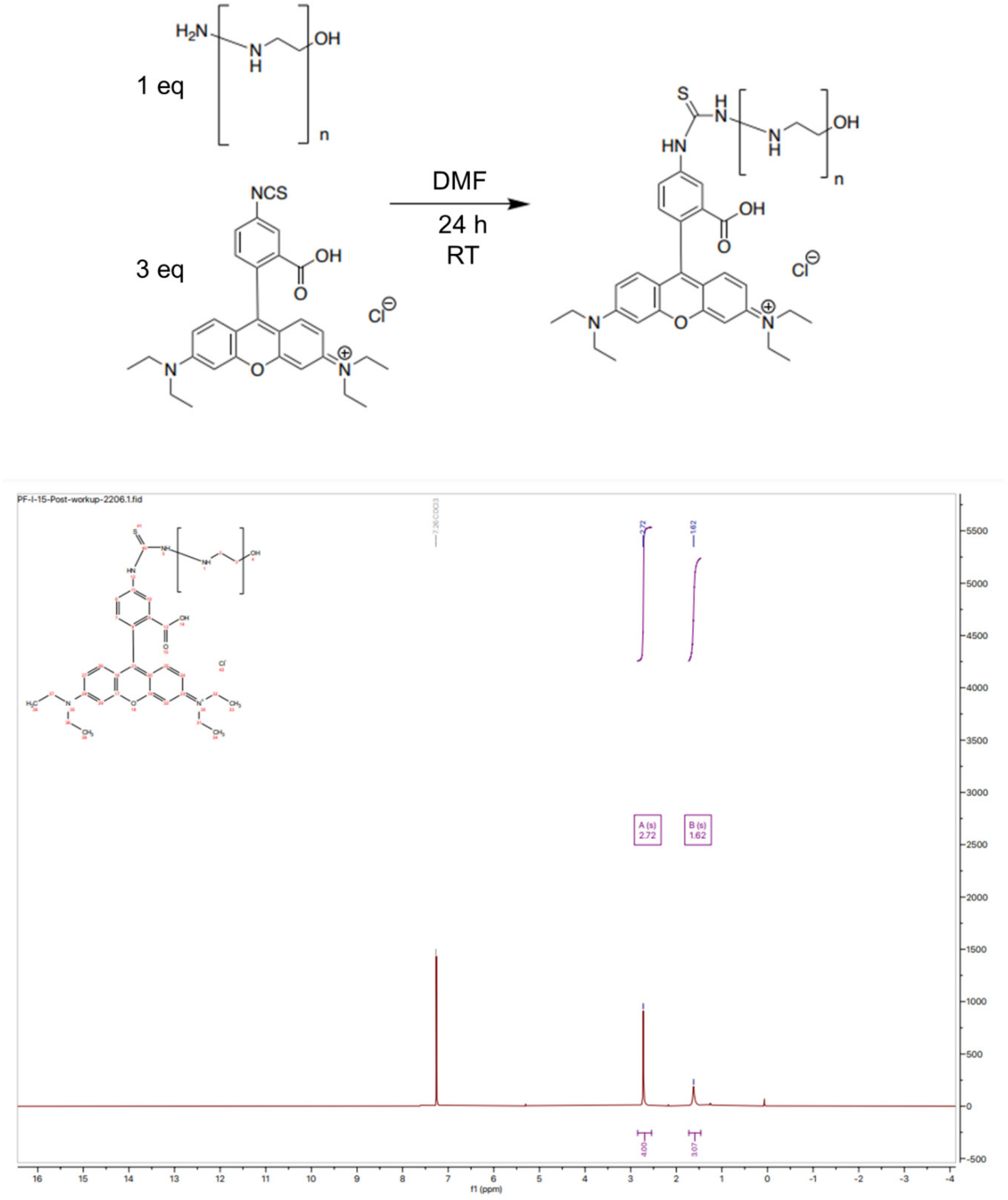

Figure S2. Reaction schematic and 1H NMR spectrum of synthesised 22 kDa L-PEI. 300 MHz, CDCl3.

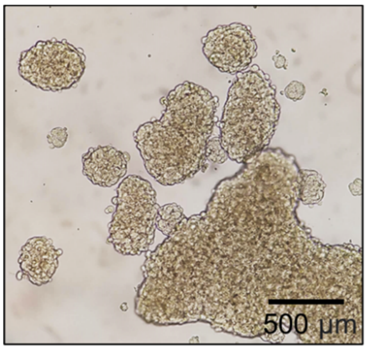

Figure S3. NCH421K spheroid cell culture. Inverted light microscope image of NCH421K spheroids grown in 96 well culture dishes for 4 days before imaging at 4× * 10× = 400× magnification using an Axio Vert A1 inverted light microscope (Zeiss) coupled to a ProgRes C5 cool (Jenoptik) camera.

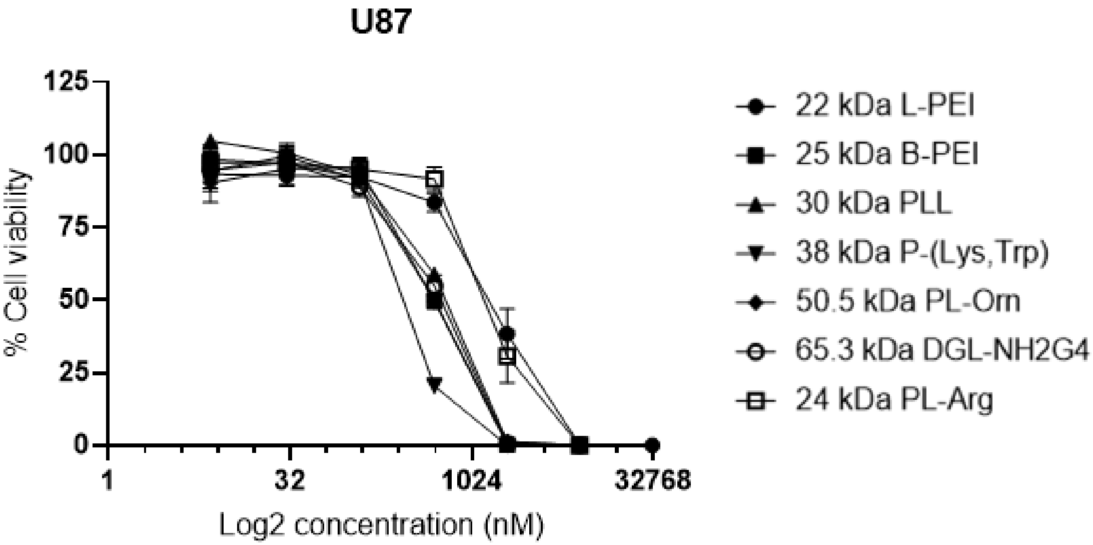

Figure S4. IC50s for the U87-MG cell line. CelltiterGlo 3D viability dose-response of 24 h treated U87 cells. Values represent the mean of at least n = 3 independent replicates. Error bars represent +/− one SEM.

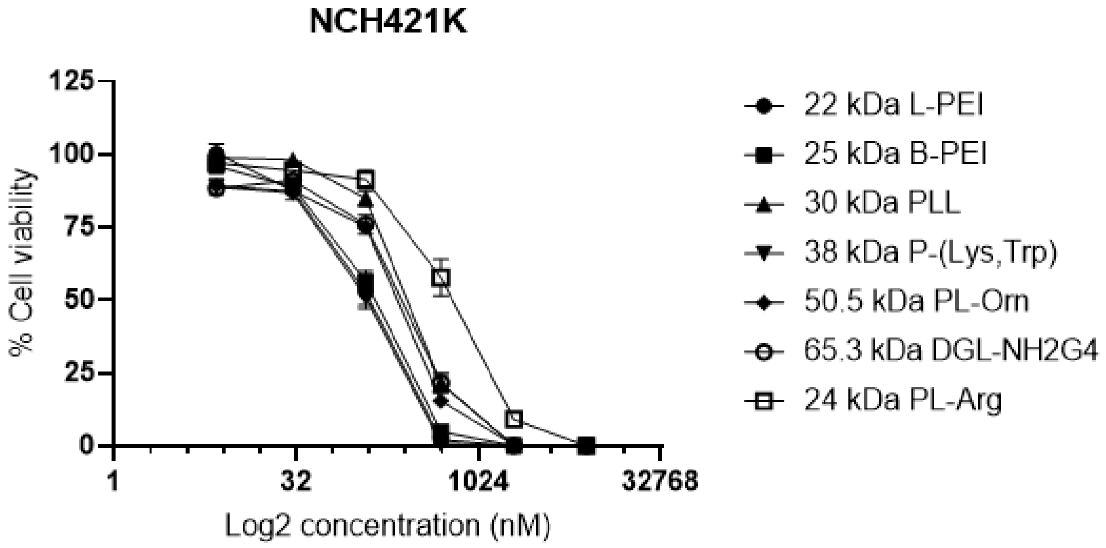

Figure S5. IC50s for the GSC cell line NCH421K. CelltiterGlo 3D viability dose-response of 24 h treated NCH421K cells. Values represent the mean of at least n = 3 independent replicates. Error bars represent +/− one SEM.

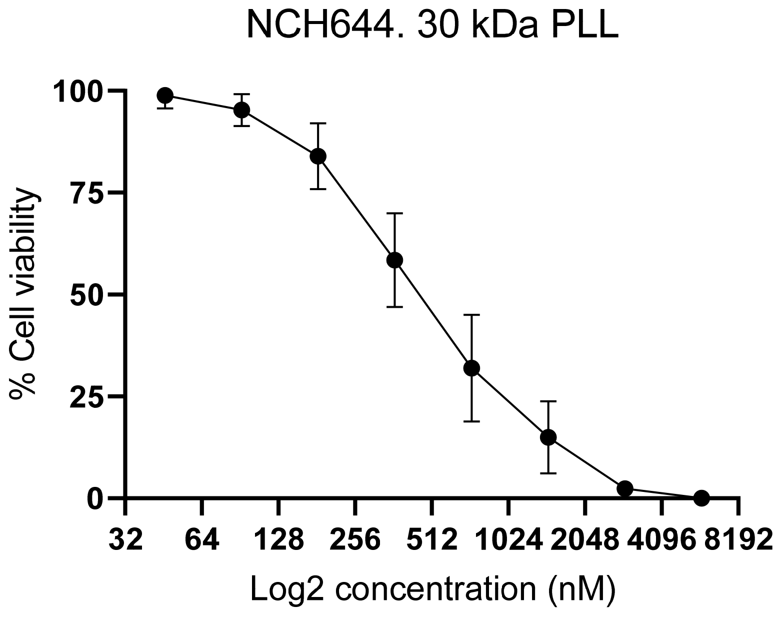

Figure S6. IC50s for the GSC cell line NCH644. CelltiterGlo 3D viability dose-response of 24 h treated NCH644 cells. Values represent the mean of at least n = 3 independent replicates. Error bars represent +/− one SEM.

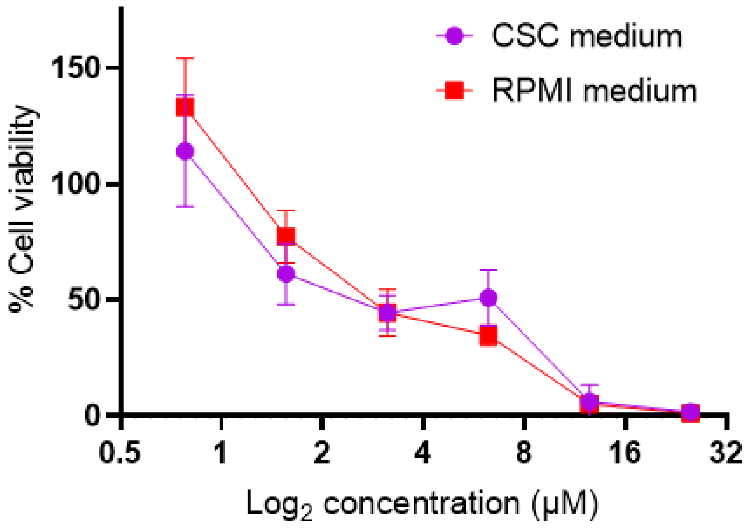

Figure S7. Lack of culture medium dependent toxicity for NHC-Ir(III). CelltiterGlo 3D viability dose-response of 24 h treated U87 cells in either their normal (RPMI) or CSC medium. Values represent the mean of at least n = 3 independent replicates. Error bars represent +/− one SEM.

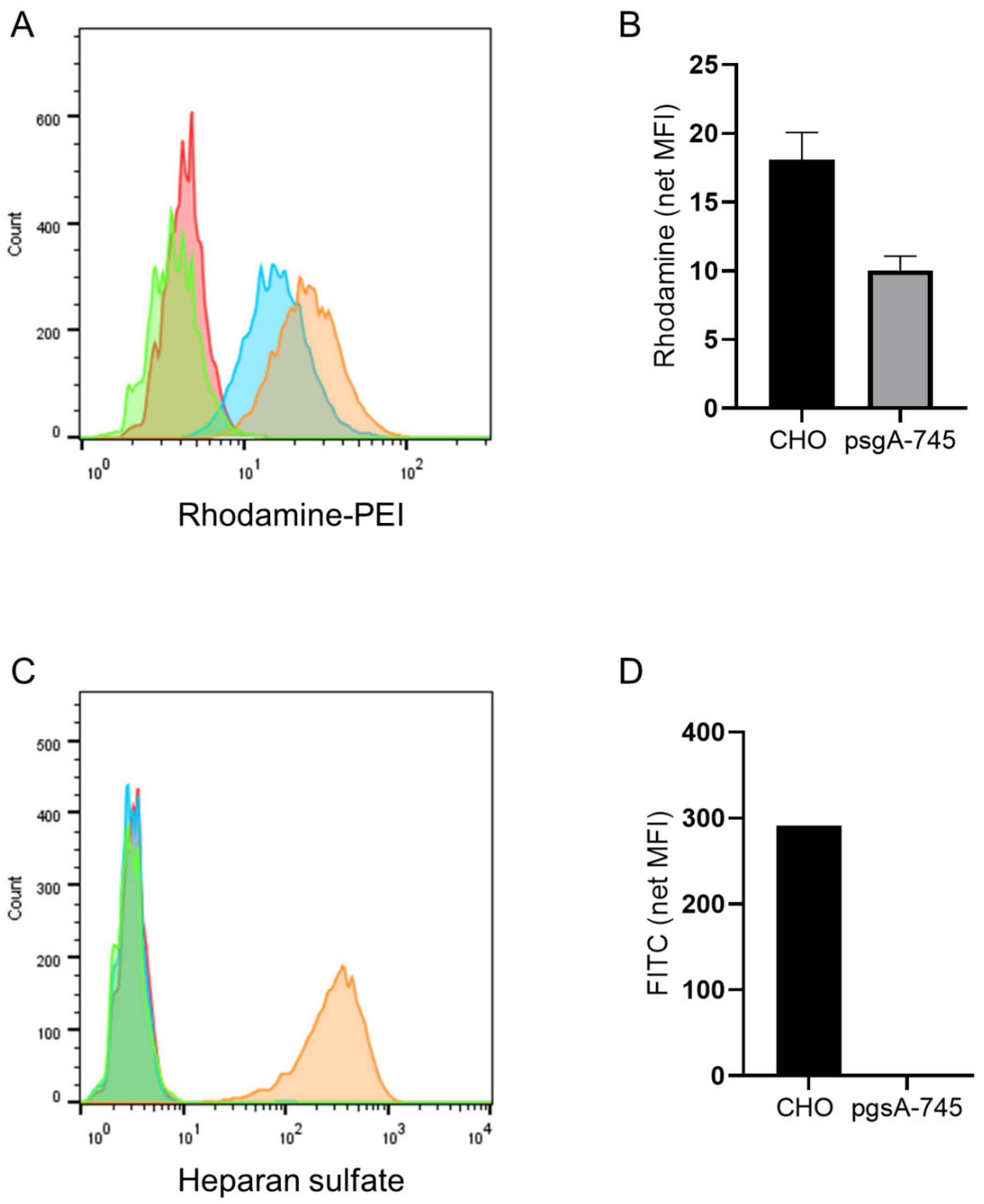

Figure S8. Reduced PEI affinity of glycosaminoglycan deficient CHO cells. (A) Representative fluorescence histograms (FlowJo) of 2 h, 37 °C Rhodamine-PEI incubated CHO-K1 WT and CHO-pgsA-745 mutant cells. (B) Bar charts of net MFI (median) (MFI stained – MFI unstained) of stained cells. Values represent the mean of n = 2 independent experiments +/− one SEM. (C) Representative fluorescence histograms (FlowJo) of heparan sulfate stained CHO-K1 WT and CHO-pgsA-745 mutant cells. (D) Bar charts of net MFI (median) (MFI stained – MFI unstained) of stained cells. Values represent one experiment. Red = Non-stained (A) or secondary antibody only (C) stained CHO cells. Green = Non-stained (A) or secondary antibody only (C) stained pgsA-745 cells. Orange = Rhodamine-PEI stained (A) or primary + secondary antibody stained (C) CHO cells. Blue = Rhodamine-PEI stained (A) or primary + secondary antibody stained (C) pgsA-745 cells.

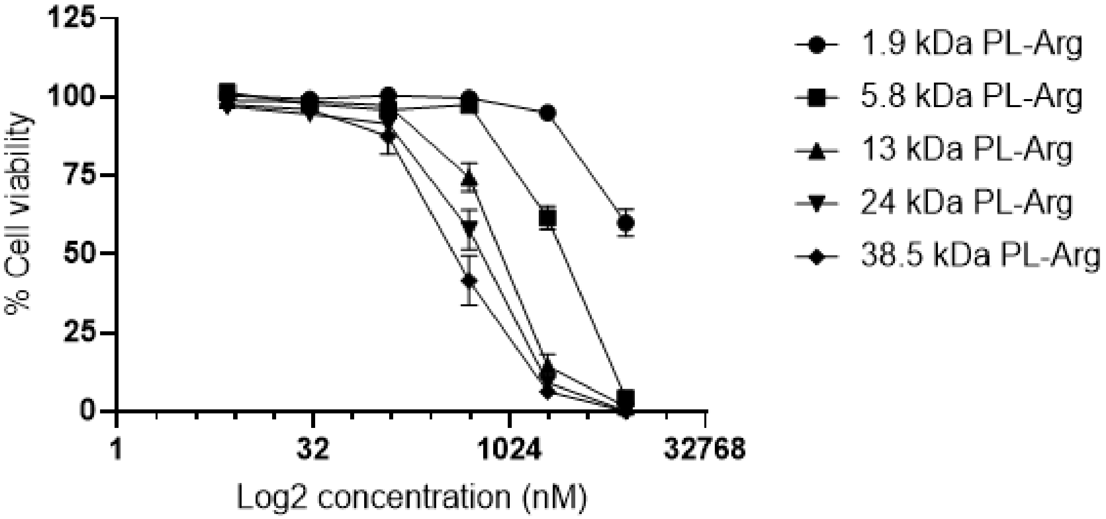

Figure S9. IC50s for the GSC cell line NCH421K. CelltiterGlo 3D viability dose-response of 24 h treated NCH421K cells. Values represent the mean of at least n = 3 independent replicates. Error bars represent +/− one SEM.

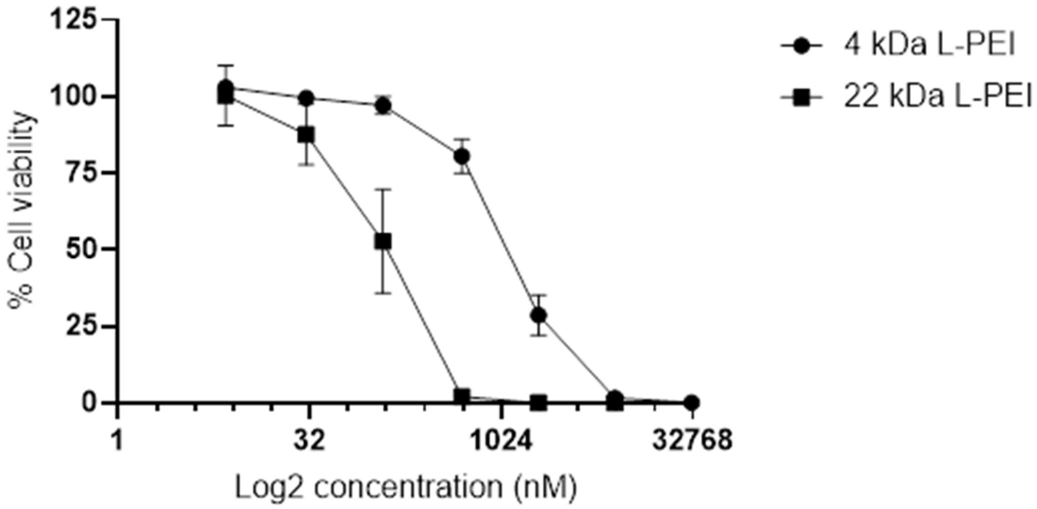

Figure S10. IC50s for the GSC cell line NCH421K. CelltiterGlo 3D viability dose-response of 24 h treated NCH421K cells. Values represent the mean of at least n = 3 independent replicates. Error bars represent +/− one SEM.
